# Using the conditioned place preference paradigm to assess hunger in dairy calves: Preliminary results and methodological issues

**DOI:** 10.1017/awf.2024.24

**Published:** 2024-04-26

**Authors:** Camille Lafon, Michael T Mendl, Benjamin Lecorps

**Affiliations:** Animal Welfare and Behaviour Group, Bristol Veterinary School, University of Bristol, Bristol BS40 5DU, UK

**Keywords:** animal welfare, dairy cattle, emotions, milk restrictions, conditioned place aversion, state-dependent learning

## Abstract

Dairy calves are typically fed restricted amounts of milk. Although feed restrictions are predicted to result in negative affective states, the relative aversiveness of ‘hunger’ remains largely unexplored in this species. Here, we investigated whether the conditioned place preference paradigm can be used to explore how calves feel when experiencing different levels of satiation. This paradigm provides insight into what animals remember from past experiences, the assumption being that individuals will prefer places associated with more pleasant or less unpleasant experiences. Sixteen Holstein calves were either fed a restricted (3 L per meal totalling 6 L per day) or ‘enhanced’ milk allowance (*ad libitum* up to 6 L per meal totalling up to 12 L per day) in their home-pen. Calves were then placed in a conditioning pen for 4 h immediately after being fed their morning meal to allow them to develop an association between the pen and their state of post-prandial satiation. Calves were conditioned across four days with their satiation state alternating between days to allow them to develop an association between pen and satiation levels. On the 5th day, calves were individually allowed to roam freely between the two pens for 30 min. We expected that calves would prefer the pen where they previously experienced higher levels of satiation, but our results show no to limited effects of treatment. However, some methodological issues (colour and side bias) prevent us from drawing strong conclusions. We discuss reasons for these issues and potential solutions to avoid these in future studies.

## Introduction

Freedom from hunger is one of the Five Freedoms for animal welfare (Farm Animal Welfare Council [FAWC] 2009). However, calves are routinely milk-restricted on dairy farms in their early life and so likely experience hunger, defined as “*a negative subjective state experienced by an animal that is chronically undernourished*” (D’Eath *et al.* 2009). Indeed, calves can consume more than 14 kg of milk per day when provided *ad libitum* access (Borderas *et al.* 2009) while the majority of farmers in the UK provide under 6 L per day (Mahendran *et al.* 2022). Later in their development, calves are further milk-restricted at weaning which involves a transition from a milk-based diet to solid feed. Weaning frequently involves an abrupt reduction in milk allowance sooner than in natural conditions (Weary *et al.* 2008).

A recent systematic review highlighted that milk restriction affects growth, health and metabolism parameters in dairy calves (Welk *et al.* 2023). For instance, when 10% of body weight in milk is offered per day, calves gained significantly less weight than those allowed up to 20% of their bodyweight (Jongman *et al.* 2020). Low milk allowances are also associated with behavioural changes, such as an increase in vocalisations (Thomas *et al.* 2001), reduced play behaviour (Groessbacher *et al.* 2020), increased competition between calves and longer time spent standing (Vieira *et al.* 2008). However, these behaviours only allow weak inferences about what calves emotionally experience when subjected to low milk allowance (Ede *et al.* 2019c). Although one recent study specifically explored the emotional impact of acute milk restriction in dairy calves, showing a diminution of calves’ cognitive performance associated with milk restriction (Lecorps *et al.* 2023), the feeling of hunger remains poorly understood in this species as well as in many others (Arbilly & Lotem 2017).

One way to assess affective states in animals is to look at what they remember from past experiences and whether they can associate a place with a more positive or more negative experience (for a review, see Tzschentke 1998). Conditioning can be used to assess an animal’s motivation to avoid or experience a stimulus (Prus *et al.* 2009). If animals are subjected to different experiences in different environments, they should prefer the environment associated with the most positive or least negative experience. This method, referred to as conditioned place preference (CPP), has been used in the past to study painful experiences such as disbudding in calves (Ede *et al.* 2019a,b; Adcock & Tucker 2020) and calves’ aversion for long-distance transportation (Creutzinger *et al.* 2022), but has yet to be applied to other emotions.

In this study, we explored whether calves would associate a specific place with different levels of satiation, and hence whether the CPP could be a useful paradigm for exploring the affective experience of hunger. To this end, calves were offered either restricted (6 L per day in two meals) or ‘enhanced’ (*ad libitum*: two meals up to 12 L per day) amounts of milk. They were placed in the conditioning pen for 4 h immediately after their morning meals of 3 L or up to 6 L, respectively. We expected that this sudden increase in milk allowance (calves were maintained on a 6-L-per-day diet before being enrolled in this experiment) would be marked enough to induce positive effects. Conditioning occurred over four days and each calf was subjected to one of the two treatments alternating across days and with the treatments taking place in different pens. On the 5th day, calves were allowed to roam freely between both pens and which pen they first entered as well as the time they spent in each pen (associated with either restricted or *ad libitum* milk) was recorded. Calves were expected to spend more time in the pen where they experienced the highest level of satiation.

## Materials and methods

The study was approved by The University of Bristol AWERB Committee (# UIN/22/020) and calves were looked in accordance with the standards of the university’s dairy farm.

### Study animals and housing

Sixteen Holstein calves (14 females and two males) were enrolled in this study (mean [± SEM] BW: 41.9 [± 3.9] kg). Within 24 h after birth, calves were separated from their dam, fed three meals of colostrum and then housed in pairs in a hutch (2.35 × 1.55 m; length × width) with an outdoor access (1.55 × 1.56 m). Before being enrolled in this experiment, calves were typically fed 6 L of milk replacer (Sprint Plus 50, Bridgmans Farm Direct, Shepton Mallet, UK; 190 Gl^–1^) per day (3 L per meal at 0830 and at 1630h) using a nipple bucket. Given that no studies have explored hunger using the CPP before, the sample size was based on the one used in previous studies using the same paradigm to assess pain in calves (Ede *et al.* 2019a). The animals used in this study were healthy calves (22.8 [± 5.5] days old) born at Wyndhurst Farm (Langford, UK). No exclusion was required during the experimental period. All calves had *ad libitum* access to water, straw, and grain and pens were bedded with more straw daily.

### Apparatus

**Figure 1.**
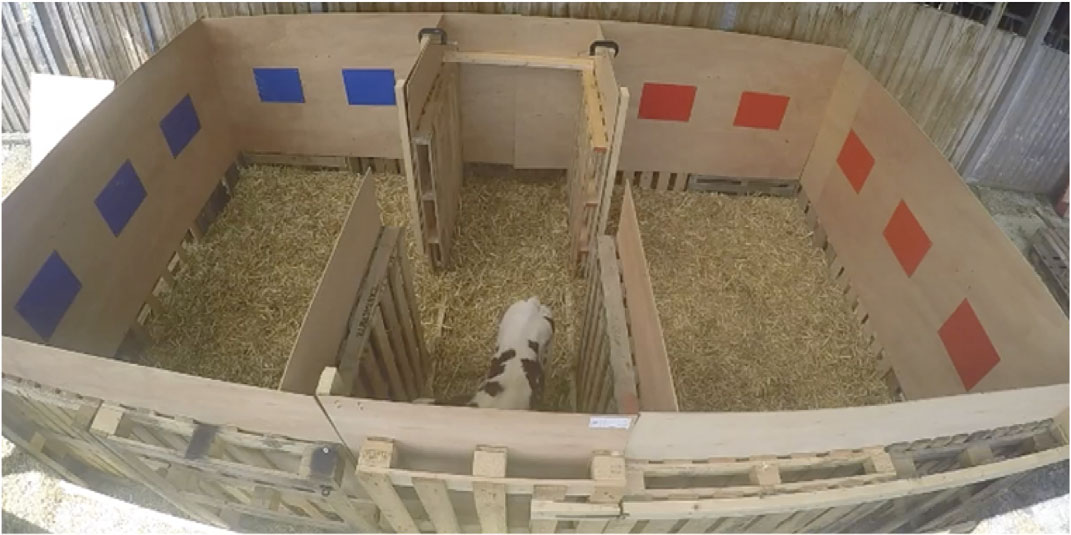
Picture of the conditioned place preference apparatus. Calves were conditioned to associate pens with either low or high satiation over four days. Immediately after the morning meal (where calves received either 3 or 6 L of milk), calves were put in one of the two pens as a pair for 4 h. Pairs of calves would always be on different colour by treatment association. On the 5th day, calves were tested individually with all three pens available for 30 min.

The apparatus was adapted from the one used by Ede *et al.* (2019a). Briefly, it was made of two identical pens (2.8 × 2.0 m) connected by a smaller central compartment (2.8 × 1.0 m). Visual cues (blue and red rectangles) were placed on the walls of the two larger pens to help calves associate pens with the different treatments (see Figure 1). Colours and material used for the panels were the same as those used for previous studies.

### Conditioning

Calves were first habituated to the arena by allowing them to explore the three pens freely as a pair for 15 min. Time spent by individual calves in each pen during habituation was recorded. This was done at around 1700h on the day preceding the first conditioning session.

Calves were then conditioned for 4 h per day over four consecutive days, immediately after the morning meal where they alternately received 3 L or up to 6 L of milk in their home-pen. Order of treatment, colour assigned and side were all pseudo-randomly allocated before calves were enrolled in this experiment. Time spent in each pen during habituation was not used to allocate calves to one or the other pen. After 4 h of conditioning in either one of the two pens, calves were brought back to their home-pen. The second meal of the day, given around 1600h, consisted of the same quantities of milk as fed at the preceding morning meal, equating to 6 L per day and up to 12 L per day for the restricted and enhanced diets, respectively.

Calves were conditioned at the same time as their pen-mate in the same pen (red or blue), but calves within pairs never received the same treatment (pre-feeding with 3 or 6 L of milk) on the same day to ensure that they were exposed to a different pen by treatment association. Colour (blue or red) and treatment order were counterbalanced among calves. In addition, to avoid a potential side bias, colours were moved from left to right so that half the animals were tested with red being on the right and the other half with red being on the left and *vice versa* for the blue pen. During testing, calves were provided with straw and water *ad libitum* and were protected from the sun.

On the morning of the 5th day, calves received 3 L of milk and were then tested individually for 30 min during which they could roam freely between the three different pens (blue, central and red). Behaviours were recorded using a top view camera (Go-PRO, Hero 4, San Mateo, CA, USA) and first entry choice, time spent in the pens as well as where the calves chose to first lie down were measured. Observers were not blind to treatment given that they fed calves before conditioning, but videos were randomised so that the observer was unaware of where each calf received each treatment when scoring behaviours even if they might have been able to recognise calves.

### Statistical analysis

Statistical analyses were run using SAS (version 9.4; SAS Institute Inc, Cary, NC, USA). The dataset and statistical codes are provided in the Supplementary material. Calf was always considered the experimental unit. Normality of residuals was verified graphically for all models used.

We first explored whether calves spent more time in one of the two pens during habituation, exploring the effect of colour (red or blue) and side (left or right) using a linear mixed model, including calves nested within pairs as random effect.

We then explored whether treatment (low satiation [3 L per meal] vs high satiation [6 L per meal]) influenced the time spent in the two coloured pens at testing using a linear mixed model. Fixed effects included order of treatment (first or second), colour of the treatment pen (blue or red), side of the treatment applied (left or right) and time spent in the pens during habituation (continuous). Calves’ identity was set as a random effect and nested within pairs. Differences in where calves chose to first enter and lie down during the test (if they did) were assessed using Pearson χ^2^ tests looking for effects of treatment, colour, and side separately.

## Results

**Figure 2.**
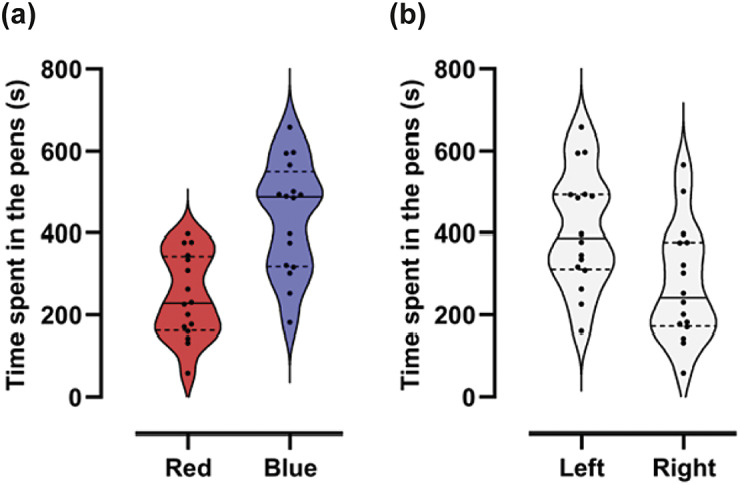
Calves (n = 16) showed a preference for the (a) blue and (b) left pen during habituation (the first time they explored the apparatus for 15 min). Calves visited the apparatus while in pairs, but data are represented at the individual level. Each dot within a boxplot represents an individual animal but each animal is represented by two points (between boxplots) due to the within-individual nature of the experimental design. Dashed horizontal lines represent quartiles and full horizontal lines represent medians.

During habituation, calves spent more time in the blue pen (Estimate [± SEM]: 194.9 [± 47.66], *F*_1,14_ = 16.72; *P* = 0.001; Figure 2[a]) and in the left pen (Estimate [± SEM]: 134.6 [± 47.66], *F*_1,14_ = 7.98; *P* = 0.014; Figure 2[b]).

**Figure 3.**
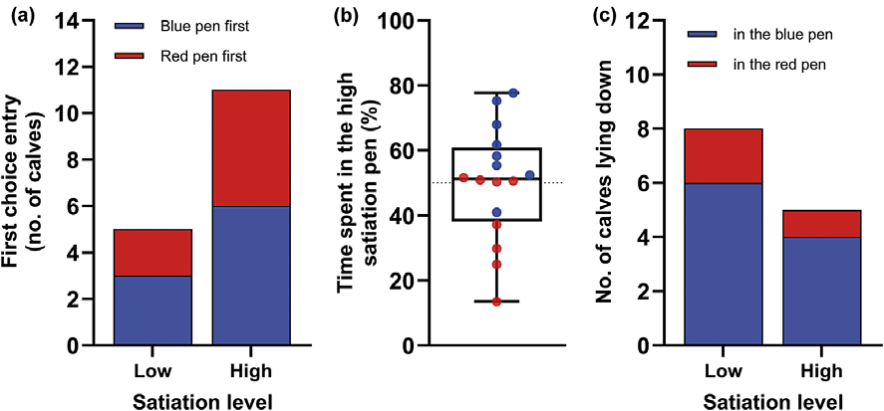
Calves (n = 16) did not (a) enter preferentially, or (b) spend more time, or (c) choose to lie down in the pen associated with higher satiation. Although colour did not affect which pen calves entered first, a strong colour preference was found with calves conditioned with the higher satiation treatment in the blue pen spending more time there at testing whilst calves conditioned with the higher satiation treatment in the red pen either showing no preference or also preferring the blue pen. Blue and red dots represent single data-points and illustrate which pen calves were trained to associate with the higher satiation treatment; likewise, calves preferred to lie down in the blue pen regardless of what treatment (low or high satiation) they associated this place with. Bars represent the number of calves that lay down in either the blue or red pen.

Although 69% (11 out of 16 calves) chose to first enter the pen where they experienced higher satiation, this distribution did not differ significantly from the null hypothesis (χ^2^ = 2.25; *P* = 0.13; Figure 3[a]), and neither colour (χ^2^ = 0.25; *P* = 0.62) nor side (χ^2^ = 0.25; *P* = 0.62) seemed to affect this choice. Although, treatment, side and order had no effects on the time calves spent in either pen at testing (after conditioning), they displayed a strong preference for the blue pen (Estimate [± SEM]: 23.0 [± 7.0], *F*_1,5_ = 10.66; *P* = 0.022; Figure 3[b]). We found no evidence that calves lay down preferentially in the high satiation pen (χ^2^ = 0.69; *P* = 0.41; Figure 3[c]), but they did prefer to lie down in the blue one (χ^2^ = 3.77; *P* = 0.05; Figure 3[c]). Side had no effect on this preference (χ^2^ = 1.92; *P* = 0.17).

## Discussion

This study explored whether CPP could provide information about calves’ affective experiences associated with receiving a larger rather than a smaller milk meal. We found limited evidence supporting this hypothesis. However, our results must be viewed with caution given that calves showed a strong preference for the blue pen both during habituation and testing and for the left pen during habituation.

Calves were expected to spend more time in the pen associated with higher milk allowance, but they did not. However, the majority of calves (eleven out of 16), chose to first enter the pen associated with higher satiation during the test, although this did not reach statistical significance. This result suggests that calves may have associated this pen with higher satiation and preferred this state, but this will need to be confirmed in future studies. The first entry is not typically measured in CPP studies in calves but may be worth being included in future studies as it is not affected by extinction learning.

The absence of conditioned place preference observed in our study can be explained by several factors. First, it is possible that calves may not experience increased milk allowance (and the assumed resultant feeling of higher satiety) as positive, but this seems unlikely given that previous studies showed that calves are motivated to obtain higher quantities of milk (Thomas *et al.* 2001; Vieira *et al.* 2008; Groessbacher *et al.* 2020).

In previous studies using CPP in dairy calves, animals experienced a stimulus directly in the conditioning pen such as disbudding (Ede *et al.* 2019b), an analgesic injection to induce pain relief (Adcock & Tucker 2020), or transport (Creutzinger *et al.* 2022). In the current study, the treatment was applied before conditioning (restricted vs enhanced diet) so that we could explore whether calves would associate a place with their level of satiation. Although cows can associate food quality with spatial location (Bailey & Sims 1998), it may not make evolutionary sense to remember and prefer places where one felt more satiated as such locations do not necessarily predict a short-term future benefit in situations where the animal has depleted the food source. It may be easier for calves to make associations between places where they receive different amounts of milk compared to places where they spend time *after* receiving different amounts of milk. If so, this would imply that a post-prandial experience of increased satiety is less positive or less easy to remember than the experience of consuming a larger meal.

Before being enrolled in this experiment, calves were fed with 6 L of milk per day. During conditioning, the amount of milk increased to 12 L per day for half of the experimental days and stayed at 6 L for the other half. Previous studies showed behavioural signs of hunger in feed-restricted calves who were otherwise fed *ad libitum* milk before experimental manipulations (Thomas *et al.* 2001), or in groups of calves who had been fed different amounts of milk since they were born (Vieira *et al.* 2008). Here, calves had previous experience with feed restrictions (they were on a 6-L diet before the experiment), which may have limited the experience of hunger in our studied population when on the standard diet. This may have reduced the contrast between the low and high satiation treatments. Moreover, Buckley *et al.* (2012) used CPP to assess broilers’ aversion for hunger and birds showed a significant preference for the pen associated with *ad libitum* feed, but only when they were hungry at testing. In our study, calves received 3 L of milk before testing and so would not have been completely unsatiated.

In addition, we could not measure individual consumption of straw, starter and water. It is possible that calves ate more starter or straw on days with the standard diet and this may have affected how satiated they felt. However, some studies have shown that it takes several weeks for calves on different allowances to display different starter intake (Frieten *et al.* 2018; Hammon *et al.* 2018; Schäff *et al.* 2018).

Some practical considerations may also have affected our results. To the best of our knowledge, this experiment was the first to condition calves in pairs (calves within pairs were not on the same treatment). This aimed to increase statistical power and reduce other confounding factors such as the effect of social isolation during conditioning. However, we chose to run tests individually because we expected calves would influence each other when given a choice between pens. Although we did not test for this during habituation, calves were often observed to follow each other. This means calves were socially isolated for the first time at testing, which may have affected our results by distracting them. Finally, calves’ preference for the blue pen during habituation and testing was unexpected and could explain why we did not detect any treatment effects. The colours chosen were not part of the calves’ environment and the experimental design was directly adapted from the one used previously to study pain (Ede *et al.* 2019a,b) and these studies did not report any colour or side biases. Our testing arena slightly differed – visual cues in the previous studies were a combination of shape and number of coloured panels (three red squares in one pen or two blue triangles in the other), while panels in our study were only distinguished by colours (blue and red).

Although they are dichromats, cows have the ability to discriminate blue and red (as evidenced by the strong bias observed here) and seem to express different behaviours under blue or red lighting, such as increased activity and reaching a familiar handler faster under red compared to blue light (Phillips & Lomas 2001). To date, there is no evidence that calves have an aversion to red colouration, which could explain pen preferences in our study. However, in the experiment by Lemos Teixeira *et al.* (2017) cows spent less time drinking and tended to have fewer sips from a red water trough compared to grey or green troughs. Side also had a significant effect on the time spent in each pen during habituation (left preference). The left pen was closer to calves’ home hutches which might explain the difference. However, the side bias was transient as it was not detected at testing but only during habituation. This could potentially be explained by several factors such as time at testing or stress during the first exploration.

Methodological challenges faced here should encourage future studies using the CPP in calves to investigate whether the experimental design is vulnerable to biases due to colour, side preferences or other factors. For instance, Creutzinger *et al.* (2022) studied calves’ response to transportation and a novelty bias was detected; calves preferred the pen in where they were not conditioned regardless of whether or not they were transported.

### Animal welfare implications

The CPP is a powerful tool for assessing animal affective experiences, but its application is limited for now in animal welfare research. This study highlights some methodological issues associated with CPP to evaluate the aversiveness of hunger and low satiation, a poorly studied but important welfare concern for many farm animal species. We suggest refinements that should help future research exploring how feed restriction affects the welfare of dairy calves and other species.

## Conclusion

We explored whether calves would associate a place with the experience of higher satiety and presumed associated positive affect. Our results do not support the idea that calves show a conditioned place preference for locations where they spend time following a larger milk meal, which may suggest the CPP is not appropriate, or should be refined, to assess this type of affective experience. However, methodological issues prevent us from drawing any firm conclusion and future studies on this species should consider that factors such as colour and side may generate biases that interfere with the results of conditioned place preference tests.

## Supporting information

Lafon et al. supplementary material 1 Lafon et al. supplementary material

Lafon et al. supplementary material 2 Lafon et al. supplementary material

Lafon et al. supplementary material 3 Lafon et al. supplementary material

## Supplementary material

The supplementary material for this article can be found at http://doi.org/10.1017/awf.2024.24.
