# Supplementary material for: Using the conditioned place preference paradigm to assess hunger in dairy calves: Preliminary results and methodological issues
Source: Anim Welf. 2024 Apr 26;33:e22. doi: 10.1017/awf.2024.24 (PMC11062768; doi:10.1017/awf.2024.24)
Supplement: Lafon et al. supplementary material 3 — Lafon et al. supplementary material [file S0962728624000241sup003.pdf]

Statistical codes to be run in SAS.

```
FILENAME REFFILE 'C:\Users\vr22128\OneDrive - University of
Bristol\Desktop\Hunger project_CPP_Summer22\Dataset_CPP_hunger_sas.csv';

PROC IMPORT DATAFILE=REFFILE
      DBMS=csv
      OUT=CPP_hunger;
      GETNAMES=YES;
RUN;

data numbers;
      set CPP_hunger;
run;

PROC CONTENTS;
RUN;

proc univariate plot normal;
var Time Prop_treat Time_hab;
      run;

Proc means data = CPP_hunger ;
var Time Prop_treat Time_hab;

** HABITUATION
*Preference for one side or one colour during habituation;
Proc mixed data = CPP_hunger ;
Class Side Pair Calf Colour Treatment ;
Model Time_hab = Side Colour Side Colour / solution residual  ;
Repeated / subject =Calf(Pair) type=ar(1);
Lsmeans  / cl diff;
Run;
*Results:
*Type 3 Tests of Fixed Effects
Effect Num DF Den DF F Value Pr > F
Side 1 22 7.98 0.0099
Colour 1 22 16.72 0.0005
Side*Colour 1 22 1.07 0.3117;
* The left and the blue were preferred

** TESTING;
Proc mixed data = CPP_hunger ;
Class Side Pair Calf Colour Treatment Order ;
Model Time = Treatment Side Colour Time_hab Order / solution residual  ;
Repeated / subject =Calf(Pair) type=ar(1);
Lsmeans  / cl diff;
Run;

*Results:
Type 3 Tests of Fixed Effects
Treatment 1 20 0.03 0.8641
Side 1 20 1.56 0.2257
Colour 1 20 9.34 0.0062
Colour*Treatment 1 20 0.01 0.9137
* Blue was preferred;
```
